# Epidemiological determinants and PCR results in Central African inhabitants with a new and frequent HTLV indeterminate Western Blot pattern exhibiting mostly p28, p32, p36, and a shifted GD21

**DOI:** 10.1186/1742-4690-8-S1-A73

**Published:** 2011-06-06

**Authors:** Claudia Filippone, Sylviane Bassot, Edouard Betsem, Sabine Plancoulaine, Sara Calattini, Antoine Gessain

**Affiliations:** 1 Unité d’Epidémiologie et Physiopathologie des Virus Oncogènes, URA CNRS 3015, Département de Virologie, Institut Pasteur, Paris, 75724 Cedex 15, France; 2 Faculté de Médecine et des Sciences Biomédicales, Université de Yaoundé I, Yaoundé, Cameroun; 3 INSERM/Université Paris 5, Unité 550, Faculté de Médecine Necker, Paris, 75015, France

## Background

HTLV indeterminate WB patterns are frequently observed in plasma/serum samples from persons living in intertropical areas.

## Material and methods

In the framework of ongoing projets on HTLV-1/2 and related viruses in central Africa, we systematically analysed by WB, plasma from villagers living in south Cameroun. The studied group included 2155 individuals (mean age 44, range 2-90, 982 women/1173 men), either Bantous (1258) or Pygmies (897). All plasma samples were tested by WB (HTLV 2-4 MPD) with interpretation done according to manufacturer instructions. Only clear bands were considered as positive/informative. DNA extracted from buffy-coat were subjected to PCR using several primer pairs known to detect HTLV-1/2/3/4. Positive PCR bands were sequenced.

## Results

Among the 2155 plasma samples, 48 were HTLV-1, 20 HTLV-2, and 134 HTLV. Furthermore, 955 were indeterminate including 100 HGIP (HTLV-I Gag-indeterminate pattern) [1], and 57 with a peculiar pattern exhibiting mostly p28, p32, p36, and a shifted GD21. The other samples were either WB negative (998) or exhibited mostly faint or unique p19 or p24 bands. Most HTLV-1 samples and some HTLV were found PCR positive. In contrast, all the others (HTLV-2, HGIP, new WB pattern and other indeterminate) were found PCR negative except in one case of a HTLV-3 infection [2]. Epidemiological determinants of the persons with this new pattern were different from those with HTLV-1.

## Conclusions

Search for the origin of this frequent new WB is ongoing with special insights concerning cross-reactivities with parasitic antigens as suggested for the HGIP pattern [3].
